# The Clinical Utility of Labial Gland Biopsy in Patients With Suspected Neuro‐Sjögren

**DOI:** 10.1002/iid3.70507

**Published:** 2026-09-23

**Authors:** Zhen Tian, Xuemei Li, Xia Li, Yajie Wang, Chunxiu Wang, Yi Zhao

**Affiliations:** ^1^ Department of Rheumatology and Allergy Xuanwu Hospital of Capital Medical University Beijing China; ^2^ Department of Pathology Xuanwu Hospital of Capital Medical University Beijing China; ^3^ Evidence‐Based Medicine Centre Xuanwu Hospital of Capital Medical University Beijing China

**Keywords:** diagnostic utility, labial gland biopsy, neurological involvement, neuro‐Sjögren, Sjögren's disease

## Abstract

**Background:**

Neuro‐Sjögren, the neurological subtype of Sjögren's Disease (SjD), is difficult to diagnose due to its heterogeneity and diverse manifestations. While the diagnostic role of labial gland biopsy (LGB) in SjD is established, its significance in Neuro‐Sjögren remains unclear.

**Objective:**

To assess the diagnostic utility of LGB in patients with suspected Neuro‐Sjögren.

**Methods:**

We conducted a retrospective cross‐sectional study at Xuanwu Hospital, Capital Medical University (2019–2023). Of 456 patients with suspected SjD, 135 were classified as suspected Neuro‐Sjögren and 277 as suspected non‐Neuro‐Sjögren. According to our center's diagnostic pathway, with reference to the 2016 ACR/EULAR criteria, patients were categorized into LGB‐required (*n* = 91) and non‐LGB‐required (*n* = 44) groups. Logistic regression identified factors associated with LGB requirement. LGB‐required patients were further stratified into LGB‐positive (*n* = 74) and LGB‐negative (*n* = 17) for clinical and laboratory comparison.

**Results:**

Suspected Neuro‐Sjögren patients demonstrated higher LGB requirement (67.4% vs. 56.3%, *p* = 0.031) and positivity (81.3% vs. 51.3%, *p* < 0.001) compared with suspected non‐Neuro‐Sjögren patients. Independent factors associated with biopsy requirement included younger age at onset and early‐onset neurological symptoms, whereas anti‐SSB positivity and ANA titers ≥ 1:320 were associated with a lower likelihood of requiring biopsy. LGB‐positive patients more frequently showed non‐specific inflammatory or immunologic abnormalities, including higher erythrocyte sedimentation rate, elevated serum IgG, and higher antinuclear antibody titers (≥ 1:320).

**Conclusion:**

LGB may provide supportive information as part of the diagnostic work‐up in selected patients with suspected Neuro‐Sjögren, particularly when serological findings are negative or inconclusive. Associations with LGB requirement should be interpreted in the context of the center‐specific diagnostic pathway and should not be regarded as universal indications for biopsy.

## 1 Introduction

Sjögren's Disease (SjD) is a chronic systemic autoimmune disorder that primarily targets the exocrine glands, causing symptoms such as dry mouth and dry eyes [1]. It may also involve multiple organ systems [2, 3]. When neurological symptoms are present, the condition is commonly referred to as Neuro‐Sjögren [4]. This subtype presents with a wide spectrum of clinical manifestations, affecting both the peripheral nervous system (PNS) and the central nervous system (CNS). The reported prevalence of neurological involvement in SjD varies between 8% and 49%, with an average of approximately 20% [5]. PNS involvement is more common, whereas CNS involvement shows variability influenced by patient characteristics and study design [6, 7, 8]. Neuro‐Sjögren may result in poor prognosis and even disability, significantly impairing patients' quality of life [9, 10]. Furthermore, patients with Neuro‐Sjögren often exhibit elevated disease activity levels, as demonstrated by higher EULAR Sjögren's syndrome Disease Activity Index (ESSDAI) scores within neurological domains [11].

Diagnosing Neuro‐Sjögren is challenging due to its heterogeneity and diverse clinical manifestations [5]. Some patients may present primarily with neurological symptoms, frequently lacking characteristic sicca symptoms or positive serological markers [12], complicating early detection [13]. Studies indicate that the average diagnostic delay for Neuro‐Sjögren ranges from 3 to 6 years, with longer delays observed in anti‐SSA (also known as anti‐Ro) positive patients or those initially presenting with neurological symptoms [14].

Labial gland biopsy (LGB) plays a key role in aiding the clinical assessment and classification of SjD, especially in anti‐SSA negative patients or those without objective glandular impairment. Although the 2016 ACR/EULAR classification criteria incorporate LGB for research classification, it is important to emphasize that these criteria are not intended for clinical diagnosis. LGB may be considered when classification remains uncertain in research settings [15], but its clinical application should be guided by individual patient context and expert judgment. Given the potential risks associated with the procedure—including local scarring, infection, or permanent numbness [16, 17]—as well as the requirement for experienced clinicians and expert pathological interpretation [18], its use should be carefully evaluated to balance diagnostic benefits against potential harms.

While the role of LGB in diagnosing SjD is well‐established [19, 20], its clinical significance in Neuro‐Sjögren remains inadequately explored. This study aimed to evaluate the potential contribution of LGB to the diagnostic work‐up of patients with suspected Neuro‐Sjögren.

## 2 Methods

### 2.1 Study Design

This cross‐sectional, retrospective study enrolled hospitalized patients with suspected SjD admitted to the Department of Rheumatology and Allergy, Xuanwu Hospital of Capital Medical University, from January 2019 to December 2023. No formal sample size calculation was performed; all eligible hospitalized patients meeting the inclusion criteria during the study period were included.

### 2.2 Study Population

#### 2.2.1 Inclusion Criteria

Adult patients (≥ 18 years) with suspected SjD and neurological involvement were included. Inclusion criteria comprised: (1) clinical evidence of CNS or PNS manifestations, assessed by a neurologist and, when available, corroborated by objective diagnostic modalities such as neuroimaging, neurophysiological studies, or cerebrospinal fluid (CSF) analysis; (2) neurological symptoms not explained by alternative diagnoses; and (3) at least one clinical encounter during which Neuro‐Sjögren was suspected.

Patients without neurological involvement, confirmed by the absence of relevant symptoms and objective findings, were assigned to the suspected non‐Neuro‐Sjögren group for comparative analysis. Diagnostic evaluations followed real‐world clinical workflows: patients positive for anti‐SSA and with objective glandular involvement were classified according to the 2016 ACR/EULAR criteria without biopsy, whereas others underwent further work‐up, including labial gland biopsy (LGB), when classification remained uncertain. Accordingly, the LGB‐required and non‐LGB‐required groups reflected the sequence and results of the diagnostic work‐up used at our center rather than distinct biological phenotypes.

In our center, patients with suspected SjD—particularly those with neurological symptoms or inconclusive serological and objective findings—are typically admitted to undergo comprehensive inpatient evaluation. This approach allows for coordinated neurological assessment, additional laboratory testing, and LGB when clinically indicated. Accordingly, all patients included in this study were hospitalized. Outpatient cases were excluded due to incomplete diagnostic work‐up.

#### 2.2.2 Diagnostic Criteria for Neurological Involvement

Neurological involvement was defined only in patients whose symptoms were confirmed by neurological assessment and, when feasible, supported by diagnostic modalities such as magnetic resonance imaging (MRI), positron emission tomography (PET), nerve conduction studies, electromyography (EMG), and CSF analysis. PNS involvement was defined as autoimmune‐mediated peripheral neuropathy, while CNS involvement included autoimmune etiologies such as vasculitis, encephalitis, or myelitis [5]. This stringent diagnostic framework aimed to minimize misclassification by excluding non‐specific or unrelated neurological symptoms that could be incorrectly attributed to SjD. Only manifestations corresponding to the neurological domains of the ESSDAI were included [11]. An exception was made for patients with biopsy‐confirmed small‐fiber neuropathy, diagnosed according to the current diagnostic criteria, a recognized condition not originally incorporated into the ESSDAI scoring system, yet increasingly acknowledged in Neuro‐Sjögren literature [6].

#### 2.2.3 Exclusion Criteria

Exclusion criteria included the presence of other connective tissue diseases (e.g., systemic lupus erythematosus, rheumatoid arthritis, systemic sclerosis), active malignancies within the past 5 years, incomplete data, concurrent active infections, or conditions unsuitable for LGB, such as local infections, coagulopathy, or previous lip biopsy.

### 2.3 Data Collection

Data were collected from electronic medical records at the time of the initial diagnosis. Demographic information (e.g., sex, age), disease duration, and age at onset were documented. Objective glandular function was assessed using Schirmer's test and unstimulated whole saliva flow rate (UWS), while subjective sicca severity was specifically assessed using the dryness domain of the EULAR Sjögren's Syndrome Patient Reported Index (ESSPRI) [21].

Laboratory data included immunological markers such as anti‐SSA and anti‐SSB (also known as anti‐La) antibodies, rheumatoid factor (RF), antinuclear antibodies (ANA), as well as complete blood count, inflammatory markers, immunoglobulin G (IgG), and complement levels (C3 and C4). For categorical analyses, elevated IgG was defined as serum IgG above the upper limit of the reference range (> 1560 mg/dL). Only three patients had IgG levels below the lower limit of normal, and reduced IgG was therefore not analyzed as a separate category.

Overall patient‐reported symptom severity, including dryness, fatigue, and pain, was assessed using the full ESSPRI score [21]. Disease activity was assessed using the ESSDAI [11], and neurological involvement was assessed using the Neurological Involvement of Sjögren's Syndrome Activity Index (NISSDAI), which reflects new or worsening neurological symptoms. This index adopts the scoring principles of the neurological domains within ESSDAI. For patients with stable neurological damage lasting more than 1 year, a score of 0 is assigned, indicating no current disease activity [22, 23].

### 2.4 LGB

Patients with negative anti‐SSA results or objective tests not indicating impaired lacrimal or salivary gland function were considered for LGB based on clinical judgment and with reference to the 2016 ACR/EULAR classification criteria for SjD [15]. Informed consent was obtained from all patients. The biopsy was performed by experienced clinicians, and tissue samples were processed and examined by pathologists [18]. The focus score (FS) was calculated by counting lymphocytic foci (≥ 50 mononuclear cells) per 4 mm^2^ of salivary glandular tissue. An FS of ≥ 1, while not diagnostic on its own, is a key component of the classification framework and may support clinical diagnosis when used in conjunction with other findings. Notably, all patients who were not subjected to LGB were anti‐SSA positive, consistent with the 2016 ACR/EULAR criteria, under which anti‐SSA positivity in combination with other objective findings may suffice for classification without biopsy [18]. In this study, “LGB requirement” was operationally defined within our center's diagnostic pathway. Patients were categorized as LGB‐required when the available serological and objective glandular findings were considered insufficient for classification according to the 2016 ACR/EULAR criteria without biopsy, and LGB was therefore included in the subsequent diagnostic work‐up. This outcome was influenced by the sequence and results of preceding investigations, clinical judgment, and local practice, and should not be interpreted as an intrinsic disease characteristic or a universal indication for LGB.

### 2.5 Statistical Analysis

Data were analyzed using SPSS version 26.0 (IBM Corp.). Continuous variables were assessed for normality using the Shapiro–Wilk test or Kolmogorov–Smirnov test. As none of the continuous variables followed a normal distribution, they are presented as median and interquartile range (IQR), and comparisons between groups were performed using the Mann–Whitney U test. Categorical variables were expressed as frequencies and percentages, with comparisons made using the *χ* ^2^ test or Fisher's exact test.

Logistic regression analyses were conducted to identify factors independently associated with LGB requirement. Variables considered clinically relevant and/or associated with LGB requirement in univariable analyses were entered into the multivariable logistic regression model, and a backward likelihood ratio (backward: LR) procedure was used to derive the final model. Anti‐SSA was not entered into the regression model because all patients in the non‐LGB‐required group were anti‐SSA positive, making anti‐SSA status collinear with biopsy requirement in our diagnostic pathway. Schirmer test ≤ 5 mm/5 min and unstimulated whole saliva flow rate ≤ 0.1 mL/min were not included in the primary multivariable model because both variables are components of the 2016 ACR/EULAR classification framework and directly contribute to the determination of biopsy requirement. Including these variables in the main model could therefore introduce circularity in the interpretation of associations [15]. A two‐tailed *p* value of < 0.05 was considered statistically significant.

## 3 Results

**Figure 1:**
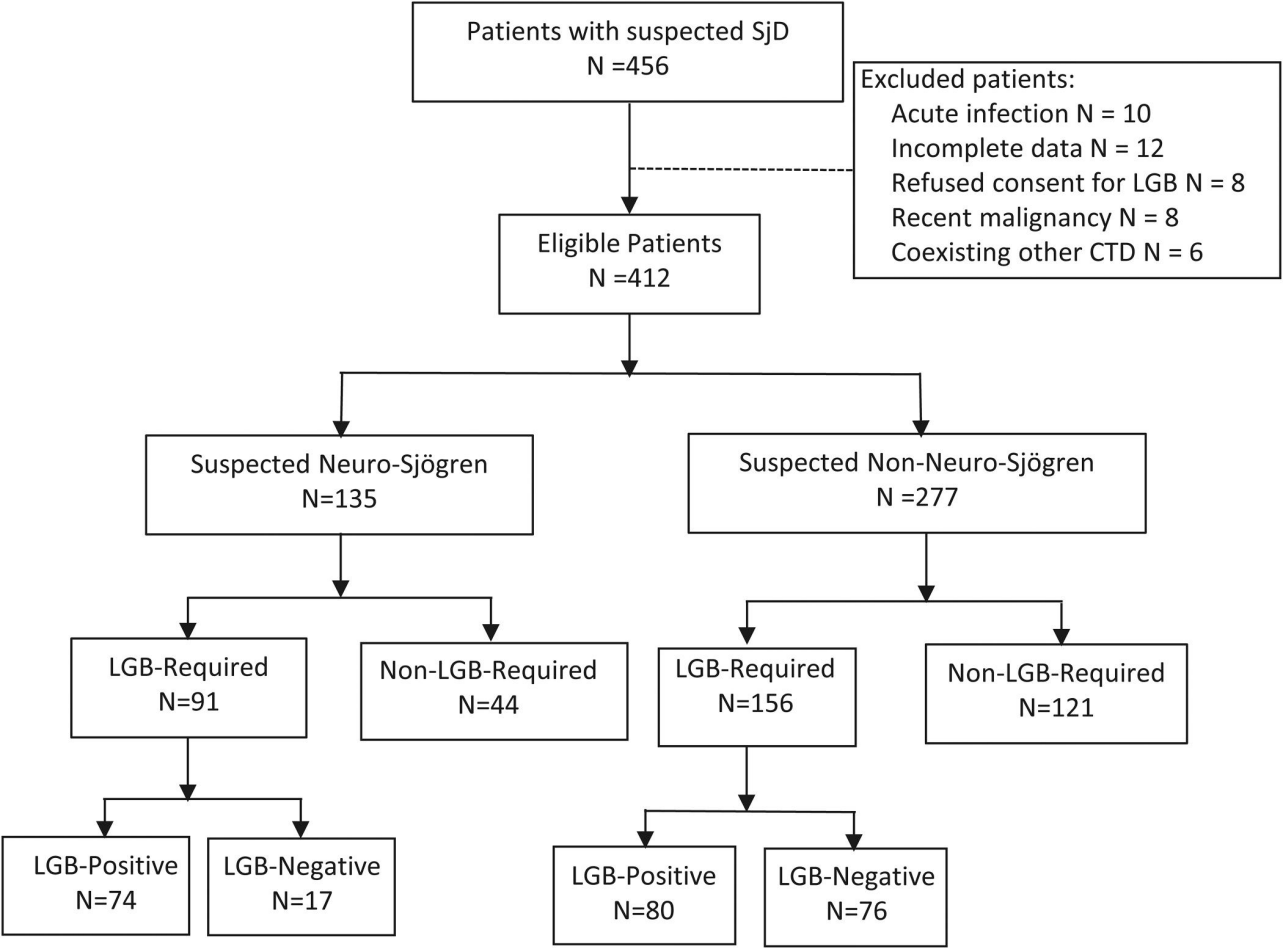
Flowchart of patient selection and grouping for suspected SjD. This flowchart illustrates the patient selection process for the study. The primary focus was on the suspected neuro‐Sjögren group (*n* = 135), while the suspected non‐neuro‐Sjögren group (*n* = 277) was included for comparison. Based on the LGB requirement as operationally defined within the study diagnostic pathway, patients were further categorized into LGB‐required and non‐LGB‐required groups. The LGB‐required group was divided into LGB‐positive and LGB‐negative subgroups. SjD: Sjögren's disease; LGB: labial gland biopsy; suspected neuro‐Sjögren: suspected SjD with nervous system involvement; suspected non‐neuro‐Sjögren: suspected SjD without nervous system involvement; LGB‐required: patients for whom the available serological and objective glandular findings were considered insufficient for classification according to the 2016 ACR/EULAR criteria without biopsy and LGB was included in the subsequent diagnostic work‐up; non‐LGB‐required: patients classified without LGB within the study diagnostic pathway; LGB‐positive: patients with a positive LGB (focus score ≥ 1); LGB‐negative: patients with a negative LGB (focus score < 1).

A total of 456 patients suspected of SjD were enrolled, of whom 412 met the inclusion criteria after screening. Among these, 135 patients with suspected Neuro‐Sjögren were identified as the primary study population. These patients were categorized into two subgroups: the LGB‐required group (*n *= 91) and the non‐LGB‐required group (*n *= 44). Within the LGB‐required group, 74 patients had positive LGB results and were classified as SjD (LGB‐positive), whereas 17 patients had negative LGB results and were therefore not classified as SjD by these criteria (Figure 1). Additionally, 277 patients suspected of having non‐Neuro‐Sjögren were included as a control group to compare LGB requirement and positivity rates.

Table 1 presents the clinical characteristics of patients in both the LGB‐required group and non‐LGB‐required group. No significant differences were observed between the two groups regarding sex (females comprised > 84% in both groups), neurological involvement type, or disease activity (*p* > 0.05). However, early‐onset neurological symptoms were more frequent in the LGB‐required group (65.9% vs. 36.4%, *p* = 0.001). Although the LGB‐required group had a younger age at onset (50 years vs. 53 years) and shorter disease duration (36 months vs. 48 months), these differences did not reach statistical significance. Furthermore, the LGB‐required group exhibited significantly lower percentages of impaired lacrimal function (Schirmer's test ≤ 5 mm/5 min, 51.6% vs. 79.5%, *p* = 0.002), impaired salivary gland function (UWS ≤ 0.1 mL/min, 34.1% vs. 81.8%, *p* < 0.001), and significantly lower ESSPRI‐dryness scores (5.0 vs. 6.0, *p* = 0.004) compared with the non‐LGB‐required group.

**Table 1:** Comparison of clinical characteristics between the LGB‐required and non‐LGB‐required groups in patients with suspected neuro‐Sjögren.

Characteristic	LGB‐required (*n* = 91)	Non‐LGB‐required (*n* = 44)	*p* value
Sex, female, *n* (%)	77 (84.6)	37 (84.1)	0.937
Age at onset, years, median (IQR)	50.0 (38.7, 56.4)	53.0 (45.0, 59.9)	0.067
Disease duration, months, median (IQR)	36.0 (12.0, 84.0)	48.0 (12.0, 120.0)	0.167
Early‐onset neurological symptoms, *n* (%)	60 (65.9)	16 (36.4)	**0.001**
Isolated CNS involvement, *n* (%)	48 (52.7)	24 (54.5)	0.844
Isolated PNS involvement, *n* (%)	39 (42.9)	15 (34.1)	0.330
Combined CNS and PNS involvement, *n* (%)	4 (4.4)	5 (11.4)	0.249
Schirmer test ≤ 5 mm/5 min, *n* (%)	47 (51.6)	35 (79.5)	**0.002**
UWS ≤ 0.1 mL/min, *n* (%)	31 (34.1)	36 (81.8)	**< 0.001**
ESSDAI, median (IQR)	15.0 (9.8, 17.0)	15.0 (11.3, 17.0)	0.244
NISSDAI, median (IQR)	12.5 (5.0, 15.0)	15.0 (10.0, 15.0)	0.458
ESSPRI, median (IQR)	4.0 (3.0, 5.0)	4.0 (3.0, 5.0)	0.834
ESSPRI‐fatigue, median (IQR)	5.0 (3.8, 8.0)	5.0 (3.3, 7.0)	0.951
ESSPRI‐pain, median (IQR)	2.0 (0.0, 5.0)	0.0 (0.0, 3.0)	**0.021**
ESSPRI‐dryness, median (IQR)	5.0 (2.0, 6.3)	6.0 (4.3, 8.0)	**0.004**

*Note:* Bold values indicate statistical significance (*p* < 0.05). ESSDAI, NISSDAI, and ESSPRI were assessed only in confirmed neuro‐Sjögren cases (*n* = 118: LGB‐positive *n* = 74, non‐LGB‐required *n* = 44); therefore, these variables were not applicable to all patients included in the initial diagnostic pathway.

Abbreviations: CNS, central nervous system; early‐onset neurological symptoms, refers to neurological manifestations occurring before the typical sicca symptoms; IQR, interquartile range; NISSDAI, neurological involvement of Sjögren's disease activity index; PNS, peripheral nervous system; UWS, unstimulated whole saliva flow rate.

Table 2 further illustrates the differences in laboratory parameters between the LGB‐required and non‐LGB‐required groups. No significant differences were observed between the two groups in hematological parameters (WBC, hemoglobin, platelets) or inflammatory markers (ESR, CRP) (*p* > 0.05). However, significant differences were found in immunological markers: the LGB‐required group had significantly lower IgG levels (1450.0 mg/dL vs. 1840.0 mg/dL, *p* = 0.001), RF positivity rate (26.4% vs. 56.8%, *p* = 0.001), anti‐SSB antibody positivity rate (11.0% vs. 38.6%, *p* < 0.001), and anti‐SSA antibody positivity rate (50.5% vs. 100.0%, *p* < 0.001) compared to the non‐LGB‐required group. Additionally, the proportion of patients with ANA titers ≥ 1:320 was significantly lower in the LGB‐required group (75.8% vs. 93.2%, *p* = 0.015).

**Table 2:** Comparison of laboratory characteristics between LGB‐required and non‐LGB‐required groups in patients with suspected neuro‐Sjögren.

Characteristic	LGB‐required (*n* = 91)	Non‐LGB‐required (*n* = 44)	*p* value
WBC, median (IQR) (thousand/μL)	4.5 (3.6, 6.3)	4.4 (3.7, 5.7)	0.901
Hemoglobin, median (IQR) (g/dL)	12.2 (11.3, 13.2)	12.1 (11.6, 13.0)	0.769
Platelets, median (IQR) (thousand/μL)	207.0 (157.0, 245.0)	220.5 (174.0, 255.0)	0.374
ESR, median (IQR) (mm/h)	14.0 (8.0, 23.0)	18.5 (9.3, 31.8)	0.117
CRP, median (IQR) (mg/L)	2.0 (1.4, 3.1)	2.0 (1.0, 4.8)	0.792
IgG, median (IQR) (mg/dL)	1450.0 (1130.0, 1770.0)	1840.0 (1335.0, 2420.0)	**0.001**
C3, median (IQR) (mg/dL)	79.0 (71.0, 94.0)	83.5 (73.0, 97.8)	0.251
C4, median (IQR) (mg/dL)	20.0 (14.0, 23.0)	19.0 (15.3, 24.0)	0.762
RF positive, *n* (%)	24 (26.4)	25 (56.8)	**0.001**
Anti‐SSB positive, *n* (%)	10 (11.0)	17 (38.6)	**< 0.001**
Anti‐SSA positive, *n* (%)	46 (50.5)	44 (100.0)	**< 0.001**
Anti‐CENPB positive, *n* (%)	9 (9.9)	3 (6.8)	0.791
ANA ≥ 1:320, *n* (%)	69 (75.8)	41 (93.2)	**0.015**

*Note:* Bold values indicate statistical significance (*p* < 0.05).

Abbreviations: ANA, antinuclear antibody; C3, complement 3; C4, complement 4; CRP, C‐reactive protein; ESR, erythrocyte sedimentation rate; IgG, immunoglobulin G; IQR, interquartile range; RF, rheumatoid factor; WBC, white blood cell count.

To further investigate the independent factors for LGB requirement, both univariable and multivariable logistic regression analyses were performed (Table 3). In the final multivariable model, younger age at onset (OR = 0.960, 95% CI 0.927–0.995, *p* = 0.024) and early‐onset neurological symptoms (OR = 3.634, 95% CI 1.563–8.449, *p* = 0.003) were independently associated with a higher likelihood of requiring LGB, whereas anti‐SSB positivity (OR = 0.277, 95% CI 0.103–0.747, *p* = 0.011) and ANA titers ≥ 1:320 (OR = 0.213, 95% CI 0.051–0.895, *p* = 0.035) were independently associated with a lower likelihood of requiring LGB. Elevated IgG was retained in the final model but showed only a borderline association (OR = 0.429, 95% CI 0.179–1.024, *p* = 0.056).

**Table 3:** Univariable and multivariable logistic regression analyses for factors associated with LGB requirement in patients with suspected neuro‐Sjögren.

Variable	Univariable OR (95% CI)	*p* value	Multivariable OR (95% CI)	*p* value
Age at onset	**0.969 (0.940, 0.999)**	**0.043**	**0.960 (0.927–0.995)**	**0.024**
Disease duration	**0.998 (0.994, 1.001)**	**0.160**	—	—
Early‐onset neurological symptoms	3.387 (1.597, 7.184)	0.001	**3.634 (1.563–8.449)**	**0.003**
RF positive	0.272 (0.128, 0.580)	0.001	—	—
Elevated IgG	**0.400 (0.188, 0.852)**	**0.018**	**0.429 (0.179–1.024)**	**0.056**
Anti‐SSB positive	**0.196 (0.080, 0.480)**	**< 0.001**	**0.277 (0.103–0.747)**	**0.011**
ANA ≥ 1:320	0.229 (0.065, 0.814)	0.023	**0.213 (0.051–0.895)**	**0.035**

*Note:* Bold values indicate statistical significance (*p* < 0.05).

Abbreviations: CI, confidence interval; elevated IgG (> 1560 mg/dL); OR, odds ratio.

We further compared the clinical and laboratory characteristics of LGB‐positive and LGB‐negative patients (Table 4). The results showed no significant differences between the two groups regarding sex, age at onset, disease duration, patterns of neurological involvement (isolated PNS, isolated CNS, or combined involvement), and exocrine gland dysfunction (Schirmer's test ≤ 5 mm/5 min, UWS ≤ 0.1 mL/min) (*p* > 0.05). Among patients who underwent LGB, the LGB‐positive group showed higher ESR, a greater proportion of elevated IgG, and more frequent ANA titers ≥ 1:320 than the LGB‐negative group.

**Table 4:** Comparison of clinical and laboratory characteristics between LGB‐positive and LGB‐negative patients (*n* = 91).

Characteristic	LGB‐positive (*n* = 74)	LGB‐negative (*n* = 17)	*p* value
Sex, female, *n* (%)	64 (86.5)	13 (76.5)	0.510
Age at onset, years, median (IQR)	50.0 (38.3, 55.9)	53.0 (39.0, 59.9)	0.323
Disease duration, months, median (IQR)	36.0 (10.0, 84.0)	36.0 (12.0, 78.0)	0.959
Early‐onset neurological symptoms, *n* (%)	48 (64.9)	12 (70.6)	0.653
Isolated PNS involvement, *n* (%)	34 (45.9)	5 (29.4)	0.214
Isolated CNS involvement, *n* (%)	37 (50.0)	11 (64.7)	0.273
Combined PNS and CNS involvement, *n* (%)	3 (4.1)	1 (5.9)	0.569
Schirmer test ≤ 5 mm/5 min, *n* (%)	39 (52.7)	8 (47.1)	0.675
UWS ≤ 0.1 mL/min, *n* (%)	25 (33.8)	6 (35.3)	0.906
WBC, median (IQR) (thousand/μL)	4.4 (3.5, 6.0)	5.0 (3.6, 7.4)	0.276
Hemoglobin, median (IQR) (g/dL)	12.2 (11.3, 13.2)	12.1 (11.4, 13.3)	0.764
Platelets, median (IQR) (thousand/μL)	203.0 (162.0, 234.3)	231.0 (144.5, 271.0)	0.381
ESR, median (IQR) (mm/h)	14 (9.8, 28.3)	9.0 (6.0, 19.5)	**0.042**
CRP, median (IQR) (mg/L)	2.0 (1.3, 3.0)	2.0 (2.0, 8.0)	0.338
Elevated IgG, *n* (%)	39 (52.7)	3 (17.6)	**0.009**
C3, median (IQR) (mg/dL)	79.5 (70.8, 96.3)	76.0 (70.0, 93.0)	0.923
C4, median (IQR) (mg/dL)	19.0 (14.8, 23.0)	20.0 (14.0, 26.5)	0.450
RF positive, *n* (%)	22 (29.7)	2 (11.8)	0.226
Anti‐SSB positive, *n* (%)	10 (13.5)	0 (0)	0.239
ANA ≥ 1:320, *n* (%)	60 (81.8)	9 (52.9)	**0.033**
Anti‐SSA positive, *n* (%)	41 (55.4)	5 (29.4)	0.053

*Note:* Bold values indicate statistical significance (*p* < 0.05).

**Figure 2:**
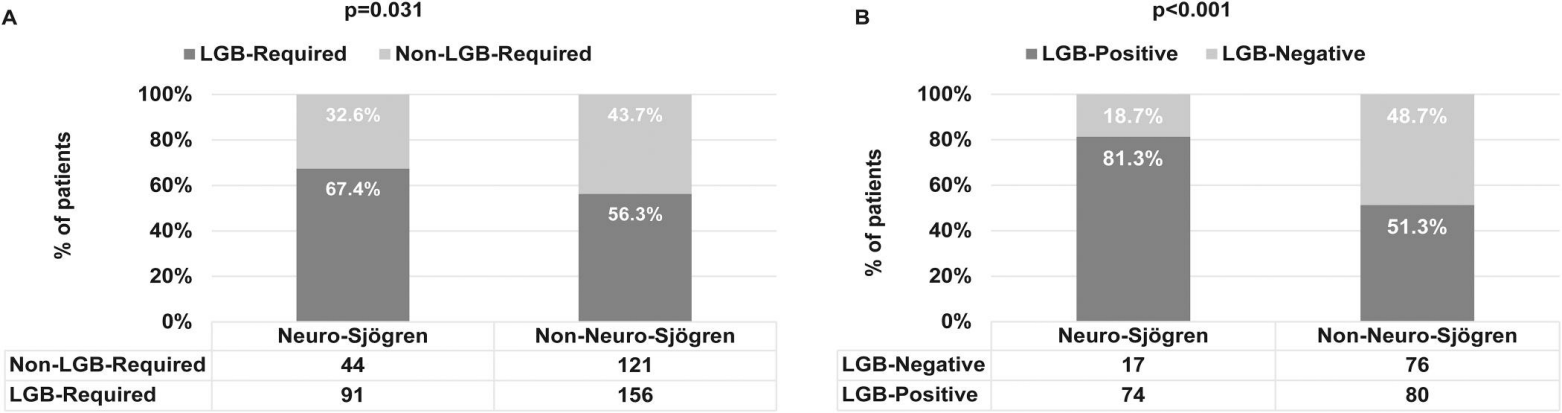
Comparison of LGB requirement and positivity rates between suspected neuro‐Sjögren and non‐neuro‐Sjögren patients. Panel (A) shows the rates of patients requiring LGB (LGB‐required vs. non‐LGB‐required) in each group, while panel (B) compares the LGB positivity rates (LGB‐positive vs. LGB‐negative) between the two groups. Statistical significance is indicated by *p* values (*p* = 0.031 and *p* < 0.001).

To further investigate the clinical significance of LGB in suspected Neuro‐Sjögren, we compared these patients with those suspected of non‐neuro‐Sjögren. The results revealed that the LGB requirement and positivity rates were significantly higher in suspected neuro‐Sjögren patients compared to suspected non‐neuro‐Sjögren patients (67.4% vs. 56.3%, *p*= 0.031, and 81.3% vs. 51.3%, *p*< 0.001) (Figure 2). Furthermore, in the suspected Neuro‐Sjögren group, the positivity rate of LGB was as high as 77.3% (33/45) among anti‐SSA‐negative patients. The remaining 12 patients, who were both anti‐SSA‐negative and LGB‐negative, were not classified as SjD based on the 2016 ACR/EULAR criteria.

## 4 Discussion

This study comprehensively evaluated the clinical significance of LGB in patients with suspected neuro‐Sjögren. Our findings show that both the LGB requirement and the positivity rates were significantly higher in suspected neuro‐Sjögren patients compared to suspected non‐neuro‐Sjögren patients. Notably, the LGB positivity rate in anti‐SSA‐negative patients with suspected neuro‐Sjögren reached 77.3%. In the final multivariable model, younger age at onset and early‐onset neurological symptoms were associated with a higher likelihood of requiring LGB, whereas anti‐SSB positivity and ANA titers ≥ 1:320 were associated with a lower likelihood of requiring LGB. Elevated IgG remained in the final model but did not reach conventional statistical significance. Moreover, among patients who underwent LGB, biopsy‐positive cases more frequently showed non‐specific inflammatory or immunologic abnormalities, including higher ESR, elevated IgG, and higher ANA titers. These findings may be compatible with a broader background of immune activation, although these markers are not specific to Neuro‐Sjögren and should not be interpreted in isolation.

Importantly, LGB requirement in this study represents reliance on biopsy within the diagnostic pathway used at our center rather than a biological characteristic of Neuro‐Sjögren. It was influenced by preceding serological and objective glandular findings, the sequence of investigations, clinical judgment, and local practice. Therefore, factors associated with LGB requirement should not be interpreted as universal clinical indications for biopsy. LGB requirement should also be distinguished from biopsy positivity: in the overall diagnostic pathway, anti‐SSB positivity and ANA titers ≥ 1:320 were associated with a lower likelihood of requiring biopsy, whereas among patients already selected for biopsy, higher ESR, elevated IgG, and higher ANA titers were more frequent in biopsy‐positive patients.

ESR and ANA are non‐specific inflammatory and immunologic markers. Unlike cryoglobulins or complement consumption, which may correlate more directly with systemic disease activity in some settings, ESR and ANA in our cohort should be interpreted only as ancillary, non‐specific indicators of immune activation rather than mechanistic markers of neurological involvement or stand‐alone indications for LGB. Among patients who had already entered a diagnostic pathway requiring histopathologic evaluation, these abnormalities may be compatible with a broader background of systemic immune activation accompanying focal salivary gland inflammation. Accordingly, these laboratory variables should be interpreted within the broader clinical context and not as specific markers of neuro‐Sjögren or as independent indications for LGB.

Previous studies have proposed the potential role of LGB in diagnosing neuro‐Sjögren. For instance, Lin et al. demonstrated that positive LGB results were associated with autonomic dysfunction [24]. Amato et al. reported that fewer than 50% of patients with ganglionopathy exhibited sicca symptoms at onset, yet more than 90% tested positive on both the Schirmer's test and LGB [25]. Moreover, among patients with small fiber neuropathy (SFN), 0.8%–30% were ultimately diagnosed with SjD, indicating a potential role for LGB in screening for SjD‐related SFN [26]. Gorson et al. emphasized that LGB can be instrumental in diagnosing SjD‐related neuropathy, particularly in patients with atypical neurological symptoms or negative antibody results [27]. Our findings suggest that LGB may contribute useful histopathological information to the diagnostic work‐up of selected patients with suspected Neuro‐Sjögren, particularly those who are anti‐SSA‐negative.

In analyzing the factors associated with the need for LGB, this study identified younger age at onset (OR = 0.960, 95% CI 0.927–0.995, *p* = 0.024) and early‐onset neurological symptoms (OR = 3.634, 95% CI 1.563–8.449, *p* = 0.003) as factors independently associated with a higher likelihood of requiring LGB. Anti‐SSA antibodies, present in all patients in the non‐LGB group, were excluded from the regression analysis because of collinearity with the decision to forgo biopsy. In addition, Schirmer test and UWS were not included in the primary multivariable model because both variables are embedded in the classification‐based diagnostic pathway used to determine whether biopsy was required [15]. Including them in the model would have introduced circularity and made the interpretation of independent associations less meaningful. In contrast, anti‐SSB positivity (OR = 0.277, 95% CI 0.103–0.747, *p* = 0.011) and ANA titers ≥ 1:320 (OR = 0.213, 95% CI 0.051–0.895, *p* = 0.035) were independently associated with a lower likelihood of requiring LGB. Elevated IgG was retained in the final model but showed only a borderline association (OR = 0.429, 95% CI 0.179–1.024, *p* = 0.056). These findings are consistent with clinical experience, as early‐stage Neuro‐Sjögren frequently lacks typical sicca symptoms or immunological markers [28]. In contrast, patients with anti‐SSB positivity or ANA titers ≥ 1:320 may already present stronger serological evidence within the overall diagnostic work‐up. This interpretation is broadly consistent with prior registry data showing that positive labial gland histopathology is strongly associated with serological features such as anti‐SSA/SSB positivity, high ANA titers, and higher IgG concentration [29]. In our cohort, such serological abnormalities may therefore reduce reliance on histopathological confirmation within the diagnostic pathway. In selected patients with younger age at onset or early‐onset neurological symptoms, especially when serological findings are negative or inconclusive, LGB may be considered as one component of an individualized diagnostic assessment.

This study also found that early‐onset neurological symptoms were associated with a higher likelihood of requiring LGB. In the LGB‐required group, 65.9% of patients presented with early neurological symptoms, which was significantly higher than the 36.4% in the non‐LGB‐required group (*p* = 0.001). This finding may identify patients whose diagnostic work‐up is more likely to include LGB, but it should not be regarded as an independent indication for biopsy or as a specific marker of Neuro‐Sjögren. Previous studies have indicated that neurological symptoms can precede classic sicca manifestations in up to one‐third of patients, often leading to diagnostic delays [7, 12, 30]. Therefore, increased clinical attention to early neurological features may help inform individualized decisions about whether LGB would add useful information in diagnostically challenging cases.

While the distribution of neurological subtypes was not significantly different between groups (PNS: *p* = 0.214; CNS: *p* = 0.273), a numerically higher LGB positivity rate was observed in patients with PNS involvement (87.2%) compared to those with CNS involvement (77.1%). Although this difference was not statistically significant, it raises the possibility that peripheral phenotypes may be more strongly associated with histopathological inflammation. Further studies are needed to determine whether PNS‐predominant subtypes reflect distinct immunopathological mechanisms within neuro‐Sjögren. Although exploratory, this possibility is broadly consistent with prior work suggesting that distinct neurological phenotypes in Sjögren's syndrome may be associated with different immunological profiles [31].

Notably, this study observed a slightly higher proportion of CNS involvement compared to PNS involvement in suspected Neuro‐Sjögren patients, which contrasts with previous studies that emphasize predominant PNS manifestations [6, 32]. This discrepancy may reflect center‐specific factors, including the neurological expertise and referral patterns at our institution. While these findings should be interpreted with caution, they highlight the importance of recognizing both CNS and PNS presentations in clinical practice. Further investigation is needed to clarify whether neurological phenotype influences the contribution of LGB to the diagnostic work‐up of suspected neuro‐Sjögren.

This study has several limitations. First, histological evaluation was limited to the focus score, without complementary analyses such as immunophenotyping or characterization of inflammatory cell subsets. Second, the retrospective, single‐center design and relatively small sample size may limit the generalizability and statistical power of the findings. Third, all participants were hospitalized patients evaluated for suspected SjD, including patients with suspected neuro‐Sjögren, and the decision to perform LGB was influenced by preceding test results, clinical judgment, and local practice. These features may introduce selection, spectrum, and indication bias and may preferentially include patients with more severe, atypical, or diagnostically complex presentations. Patients with milder or more typical disease managed in outpatient settings may therefore be underrepresented. Accordingly, the frequency of LGB requirement and the associated factors observed in this cohort may differ across institutions and diagnostic settings and should not be generalized as universal indications for biopsy. Future prospective, multicenter studies incorporating broader patient populations and comprehensive histological profiling are needed to validate and further explore these findings. In addition, the laboratory variables associated with biopsy positivity in this study, such as ESR and ANA, are non‐specific and should not be interpreted as disease‐specific markers of neuro‐Sjögren. Furthermore, although the final multivariable model was restricted to a limited number of clinically relevant variables, the number of outcome events relative to the number of model parameters remained modest, which may increase the risk of model instability and overfitting. Therefore, these associations should be interpreted with caution and confirmed in larger prospective cohorts.

In conclusion, LGB may provide supportive histopathological information as part of the diagnostic work‐up in selected patients with suspected neuro‐Sjögren, particularly when serological findings are negative or inconclusive. Younger age at onset and early‐onset neurological symptoms were associated with a higher likelihood of LGB requirement, whereas anti‐SSB positivity and ANA titers ≥ 1:320 were associated with a lower likelihood. These associations reflect the diagnostic pathway used in this study and should not be interpreted as intrinsic disease characteristics or universal indications for biopsy. Among patients who underwent LGB, biopsy‐positive cases more frequently showed higher ESR, elevated IgG, and higher ANA titers, although these markers are non‐specific and should be interpreted cautiously within the overall clinical context. Prospective multicenter studies are needed to validate the generalizability of these findings.

## Author Contributions

**Zhen Tian:** methodology, investigation, data curation, formal analysis, writing – original draft, writing – review and editing, conceptualization. **Xuemei Li:** resources. **Xia Li:** methodology. **Yajie Wang:** resources. **Chunxiu Wang:** formal analysis, supervision. **Yi Zhao:** writing – review and editing, conceptualization.

## Ethics Statement

The study was approved by the Ethics Committee of Xuanwu Hospital of Capital Medical University (Approval No.: Lin Yan Shen [2024] No. 055‐001) and conducted in accordance with the principles outlined in the 1964 Declaration of Helsinki and its subsequent amendments.

## Data Availability

The data that support the findings of this study are available within the article. Further information is available from the corresponding author upon reasonable request.
